# Early positive approaches to support for family carers of young children with developmental disabilities: adaptation and piloting in Quebec public services

**DOI:** 10.3389/fresc.2025.1627502

**Published:** 2025-11-07

**Authors:** Mélina Rivard, Mélina Boulé, Marjorie Morin, Nadia Abouzeid, Céline Chatenoud, Diane Morin, Catherine Mello, Nick Gore, Jill Bradshaw, Richard Hastings

**Affiliations:** 1 Département de psychologie, Université du Québec à Montréal, Montréal, QC, Canada; 2 Faculté de psychologie et des sciences de l'Éducation, Université de Genève, Genève, Switzerland; 3 Psychology Department, Pennsylvania State University – Berks, Wyomissing, PA, United States; 4 Department of Social Policy, Sociology and Criminology, University of Birmingham, Birmingham, United Kingdom

**Keywords:** developmental disabilities, families, intervention, participatory research, parents, program evaluation, feasibility

## Abstract

**Background:**

This paper presents the participative research undertaken to adapt and pilot the Early Positive Approaches to Support (E-PAtS) program, originally developed and evaluated in English for use in the United-Kingdom, for implementation within Québec’s public health and social services. E-PAtS supports family carers of young children with developmental disabilities by promoting their well-being and adjustment early in their services trajectory.

**Method:**

The program was translated into French and iteratively adapted based on feedback from six pilot cohorts conducted across four diverse clinical settings: a rural service center, an urban center, a specialized pediatric hospital, and a diagnostic clinic. These sites were selected to ensure demographic and geographic representativity of Québec’s population, and participating families also reflected a range of backgrounds. The adaptation process was grounded in community-based participatory research principles, actively involving parents, practitioners, managers, and researchers. Changes to the program’s content and delivery were made according to partner recommendations. Evaluation focused on social validity, effectiveness, feasibility, and fidelity of implementation.

**Results:**

Participating parents completed questionnaires and interviews, reporting improved well-being and greater confidence in self-care, indicating the program’s relevance and positive impact. Fidelity of implementation was assessed using the E-PAtS fidelity checklist, and feasibility was evaluated through session attendance logs. Both indicators were considered strong, despite the challenges posed by the COVID-19 pandemic.

**Conclusion:**

Findings support the adapted E-PAtS program’s suitability for Québec’s public services, with further refinements recommended for broader dissemination. This study highlights the value of participatory approaches in adapting evidence-based interventions across cultural and service delivery contexts.

## 1 Introduction

Early childhood is a pivotal period for children and families, marked by immense learning potential and heightened vulnerability (1, 2). Early, rapid, and coordinated access to screening, assessment, and interventions is essential for children at higher developmental risk, such those suspected of having developmental disabilities [DD^1^ (4)]. Families of children with DD have service needs that arise very early as they face increased barriers in accessing social opportunities, heightened caregiving responsibilities and stress, and additional daily challenges [e.g., socioeconomic difficulties, complex medical conditions; (5, 6)].

In Quebec, the French-speaking province of Canada where the present study took place, 25% of children start school with at least one developmental delay and without having received services (7). As in other provinces and countries, access to early interventions and family supports is restricted and delayed (8–10). This is in part due to a lack of resources within the province's public health and social services system, but also to the scarcity of intervention program options that adequately target family carers' needs (9). Canadian families may wait up to two years to obtain a formal diagnosis after first identifying areas of concern in their child's development, and then up to three more years to receive early intervention services (10). Critically, parenting stress increases proportionally with wait times for services after receiving a diagnosis (45). There is a pressing need to develop initiatives that enable earlier and more effective interventions with this population. To date, however, no program within Quebec's public services system specifically targets the psychological well-being of family carers of children with DD or family adjustment more broadly (9).

Another challenge experienced by families is the sociodemographic disparities and inequities related to DD diagnoses, which affect the availability and accessibility of interventions (11, 12). For instance, early intervention options for children with an autism diagnosis are relatively more structured and better implemented within public health and social services compared to services for other DDs (13–16). There is growing concern about the relevance of service delivery systems that are exclusively diagnosis-based, i.e., where access to services depends on obtaining a specific diagnosis (primarily autism) and is often delayed due to wait times for a diagnosis (14, 15, 17, 18). Studies increasingly highlight the relevance of interventions that transcend diagnostic categories and, rather, address service needs shared by young children with diverse clinical profiles and their family carers [e.g., support in managing behaviors that challenge (or challenging behaviors), communication difficulties, or adaptive behavior and psychological adjustment issues].

**Figure 1:**
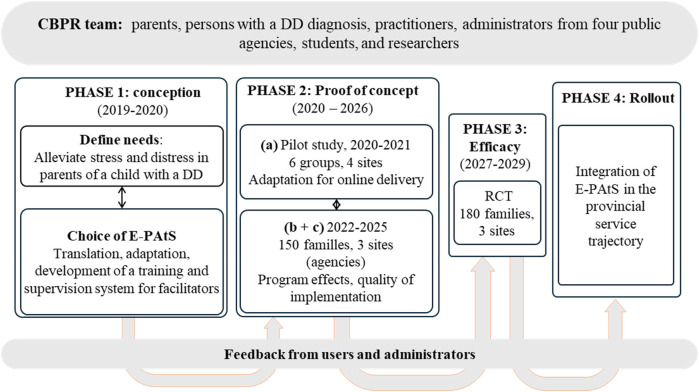
Iterative evaluation plan for E-PAtS services in Quebec. DD, developmental disabilities; RCT, randomised controlled trial.

A large, community-based participatory research [CBPR, see (19)] project was recently initiated to address these service gaps and to provide support as early as possible to family carers of children who were either recently diagnosed or awaiting a diagnosis within Quebec public agencies. The CBPR foundation of this initiative involves family carers, clinicians and administrators from within the public health and social services system, and researchers working together to co-select, -adapt, -deploy, and -evaluate a transdiagnostic intervention focused on the well-being and adjustment of family carers of children with DD during the early years. In order to meet the scientific requirements expected for the evaluation of a complex behavioral intervention in the health and social care services, this large research initiative followed a systematic approach (46, 47) that proposes a planned sequence of phases. This ensures that successive studies are carried out strategically to inform the gradual deployment and readiness of the intervention for a sustainable implementation within routine services. The planned progression began with a first phase of research targeting the selection and adaptation of the novel intervention to the new sociocultural and service delivery environments and will conclude with a fourth phase of a large-scale implementation of the intervention in routinely services the public system (see Figure 1). In the first phase of the project, the Early Positive Approaches to Support [E-PAtS; (20)] group program was selected by our CBPR team as meeting identified clinical needs (for e.g., a transdiagnostic approach and strategies to address wait times and access inequities) and its approach consistent with research on families in the context of DD [e.g., group format, psychological support to parents during early childhood; see (21)].

E-PAtS was co-developed by parents and professionals and is the focus of ongoing rigorous evaluation in the United Kingdom [U.K.; see (22)]. E-PAtS is an 8-week support group program designed for families of children aged 0–5 years with a wide range of intellectual and developmental disorders, service needs, and socio-economic backgrounds [for a detailed logic model, see (20)]. Sessions are co-delivered by two facilitators, a trained parent carer and a practitioner, who work collaboratively to support up to twelve families. These working partnerships and co-production with family carers are key components of E-PAtS. The first two sessions focus on the development of a family support system and on parents' emotional and well-being needs. Subsequent sessions of the program provide more in-depth guidance and reminders on these subjects. Session 3 is about sleep, Session 4 focuses on social interactions and communication, and Session 5 focuses on the development of adaptive behaviors. Sessions 6 and 7 build on the previous sessions and focus on support for behaviors that challenge. Session 8 is an integrative session that provides an overview of learnings and closure with co-facilitators. E-PAtS group processes aim to create a system of emotional and social support that encourages engagement and meets the well-being needs of family carers. E-PAtS invites the participation of two family carers from the child's daily life. A core design principle of E-PAtS is its adaption for local delivery. Consequently, it was designed to smoothly integrate adaptations and ensure local and cultural fit. Implementing organizations are expected to embed and update information about locally available services and systems into the curriculum and to adopt language, phrasing, and examples suitable for participating families.

Our CBPR team formalized a collaboration with the U.K.-based researchers who developed E-PAtS and built a larger team including academic researchers, clinicians, and parents to act as designers in the adaptation of the curriculum and as co-facilitators and researchers. We then proceeded with the translation and adaptation (with parents, clinical practitioners, and administrators) of the intervention and research materials and tested this French translation with a group before beginning the pilot study in winter 2020. The present study, which represented the first stage of the second phase (i.e., phase IIa in Figure 1) aimed to refine and evaluate the program in the Quebec context and thus to document, on a small number of participants, its feasibility and social validity and stakeholders' perceptions of the research process before conducting future larger trials. Thus, the present article describes the French translation and adaptation of the E-PAtS intervention for implementation within the Quebec public health and social services system, specifically in diagnostic clinics and centers providing early childhood services and specialized services for DD. This phase of the research project aimed to ensure the program's alignment with the unique characteristics of the Quebec context and the identification of practical barriers and levers inherent to the complex and resource-limited settings of public agencies. It also provided an opportunity to evaluate the social validity of the intervention from the perspective of the families it seeks to support.

### 1.2 Objectives

This paper presents the processes involved in tailoring the E-PAtS program for Quebec, assesses its feasibility for delivery within public services in the province, and examines the experience of family carers who participated in the first pilot of this project. Adaptation studies represent a crucial, initial step to guide the implementation of an intervention in a new cultural context and, ultimately, ensure its high-quality deployment in real-life settings (23). These studies document the degree to which an innovation is modified in the process of its adoption and implementation, address the intervention's feasibility within a new clinical setting, and explore potential impacts (e.g., through preliminary user feedback). Adaptations studies generally follow a process that includes understanding the community, identifying intervention needs, training staff, and modifying the program. To guide adaptation, key factors typically considered are the feasibility of the intervention (including adherence and fidelity), barriers and facilitators to implementation, social validity [including perceptions of the adequacy of the program in meeting users' needs, the acceptability of the procedures, and the perceived effects, e.g., (19)], and trends in desired behavioral outcomes.

As such, this study aimed to answer the following questions:

1. What steps, changes, and processes are involved in translating and adapting E-PAtS for the Quebec context?
2. How feasible is this intervention in terms of participants' retention and adherence, participation barriers and facilitators, and intervention fidelity?
3. How do family carers perceive the social validity of the program, specifically in terms of its ability to meet their needs, the acceptability of its procedures, and its perceived effects?

## 2 Method

### 2.1 Program adaptation

The adaptation process adhered to established practices in adaptation studies as outlined in a scoping review by Escoffery et al. (23) such as engaging stakeholders; assessing community and settings needs; selection of the program; training staff; assessing the need for adaptation; adapting program context, content, and delivery (preparing the materials); implementing (and supervise the staff) the program; and evaluating program feasibility and perceived impacts by users while continuously adapting the program. As described, the first step of this project involved creating a team based on CBPR principles (19) to ensure the active involvement of key stakeholders, namely parents, practitioners, administrators, and academic researchers (students and faculty), at every stage of the process. CBPR studies on families' needs and service centers were conducted to better understand the types of services required and to guide the selection of the program [see (9, 14, 21, 24–26)]. In 2019, E-PAtS was selected in consultation with experts and then a formal collaboration was established with the researchers who developed the intervention in the U.K., resulting Quebec-U.K. larger CBPR team. Together, they assessed community needs and the context of existing services to strategically plan the initial phase of program adaptation.

The Quebec research team (including parents, clinicians, administrators, and researchers) was trained by the original authors of E-PAtS in February 2020. Translation and planned adaptations to the E-PAtS program were made to align with a new community setting, address the specific needs of the target population, and integrate cultural aspects of the local context. We translated and adapted the language of the E-PAtS protocols and materials using a combination of lived experience and local professional expertise with continuous, concurrent verification and discussion between the U.K. and Quebec teams. The guiding principle was to maintain the integrity of the original materials, curriculum, and the nuances of phrasing and meaning, while adopting terms and expressions that reflected the local cultural context. This principle was maintained throughout the process to ensure that the program remained relevant and effective in different contexts. These adaptations were both proactive (i.e., planned) before the delivery of the first group and then reactive and based on data collected with each cohort. Table 1 details these adaptations (see also the Results section for the observations that prompted responsive adaptations).

**Table 1:** Planned and reactive adaptations of E-PAtS to the Quebec context and for online delivery.

Type	Focus	Rationale and process
Planned adaptions: Initial, in-person delivery		
Context	Population. Extending age range to include carers of children aged under 7 years	It was decided, in agreement with parents, practitioners, and administrators of the public health and social services network, to extend the age range for inclusion in E-PAtS (originally: 0–5 years) to correspond to the period of eligibility for early childhood services in Quebec, which is 0–6 years 11 months.
Context	Settings. Delivery through the public health and social services system	The E-PAtS program was implemented and evaluated directly in the public system of Quebec for potential integration into its regular service offerings. This included two public agencies, a specialized children's hospital, and a community-based diagnostic clinic. Regular staff in these community-based settings were trained directly and implemented the program as part of routine services, integrating it into the interventions offered.
Content	Translation of the material into Canadian French	An essential step in preparing to offer the program within public services was its translation into Canadian French to ensure its linguistic and cultural accessibility. The translation of E-PAtS content was iteratively carried out by multiple members of the research team (including clinicians, parents, and a manager). The Canadian French version of the program was then presented to an independent family carer (a parent) to validate the first iteration of the translated and adapted E-PAtS materials. This step allowed for refinements in language, terminology, and visuals based on the parent's feedback.
Content	Adjustments to Session 1 to include location-based services	A profile of the services available at each of the partner sites and in their respective regions was created in collaboration with the E-PAtS facilitators and site administrators. This adaptation of Session 1 is planned as a core part of the EPAtS program for all implementation sites. Additionally, the adaptation of available resources must be carried out at each new delivery site and evolve over time to account for the current services available to parents in real time.
Content	Cultural adaptations for examples and cultural references in the program.	Some of the content program and the slideshow presentations for parents was modified in minor ways to enhance its cultural relevance (e.g., public figures with a diagnosis of DD, examples, cultural references). For example, changing the positive examples of autistic people in Session 2 to include examples known to families in Quebec. Another example is that we incorporated local terminology specific to the province's healthcare and social service systems.
Delivery	Deliverer. Addition of an “observer,” separate from the E-PAtS facilitator dyad, to provide technical support	The Quebec adaptation of E-PAtS systematically includes a third person in the delivery of the intervention to support the E-PAtS facilitators in, e.g., completing the fidelity checklist (which is a component of the program and not just for research purposes) and to assist families in participating in the program. This person, referred to as the “observer,” typically helps with scheduling meetings, managing communications with parents regarding organizational aspects, and screening for additional support needs. The observer attends each session of the program and can be contacted by participating parents in case of questions or additional needs.
Delivery	Deliverer. The trained practitioner facilitators are employees of the public social and health system.	Develop a workplace training system to ensure the sustainability of the program within public services.
Planned adaptations: Online delivery in response to the Covid-19 pandemic		
Delivery	Mode. Online delivery on Zoom or Teams platform.	The Covid-19 pandemic led both the U.K. and Quebec teams to make adaptations to offer E-PAtS online through platforms available via service networks and/or the researchers' university.
Delivery	Deliverer. Additional roles of the observer.	There was an observer at each remote session, with the camera turned off. This observer provided technological support to participants over the phone and online as needed (including assistance with digital technology).
Delivery	Reducing group size	Group sizes were reduced from 12 to 4–6 families to help manage online interactions.
Content	Reduction of the duration of a session from 2 h 30 to 2 h to promote engagement and participation while being on an online platform.	Sessions were shortened in acknowledgement of the fatigue associated with online interactions and the fact that some discussions may be briefer when online.
Content	Addition of Session 0 to introduce the online modality	Introductory session were added to help participants feel comfortable online and make sure all are able to use the online platform
Content	Changes in materials and exercises.	Additional considerations for how best to present materials and exercises online (i.e., use of breakout rooms for group activities).
Reactive adaptations: Response to ongoing program evaluation		
Content	Removal of explanatory videos for Sessions 3, 4, 5, and 7.	These videos were not available in French and their simultaneous translation was not well-received. These were replaced by personal examples from the parents facilitators.
Content	Adding more content on available services and on the pathway to accessing these services	We included the presentation of a portal in Quebec that directs parents toward relevant resources and information. During Session 1, E-PAtS facilitators help parents filter the information and services based on their situation (child's age and diagnosis or needs, region of residence).
Delivery	Change in the mode of transmission of the material to parents.	Because the online learning platform was not deemed a user-friendly method of sharing the intervention materials, the observer emailed (or, by request, mailed a physical copy of) materials directly to parents before each session.

The first cohort was initially scheduled to begin in April 2020. Thus, planned adaptations of E-PAtS for the Quebec context began in February 2020. However, due the lockdown measures implemented in March 2020 in response to the Covid-19 pandemic, the intervention, delivery, and research approach required rapid adaptations. Modifications for online (as described in Table 1) delivery of the intervention were made from March to August 2020 in both Quebec and the U.K.; the first online cohort in Quebec began in September 2020.

### 2.2 Design and settings for piloting and continuous adaptations

E-PAtS was implemented over six consecutive cohorts to address Questions 1 (reactive adaptations), 2 (feasibility), and 3 (social validity). A single arm pre-experimental pre-test post-test design (27) was adopted along with continuous observation measures and individual qualitative interviews after the intervention and three months later. The intervention was conducted in four different establishments: two public health and social services agencies (one in Montreal, the largest city in Quebec, and one in the suburbs of Montreal), a specialized children's hospital, and a community-based diagnostic clinic for DD.

### 2.3 Participants: family carers

A total of 31 family carers were recruited to participate in E-PAtS (28 mothers, 3 fathers) and divided into six cohorts. Two carers per family were invited to integrate E-PAtS groups as planned in the E-PAtS program, however only one parent per family participated (see Results for more details). Table 2 describes the characteristics of participating households. The majority of participating families reported living in an intact, two-parent family situation (70.6%), with fewer reporting being separated or divorced (11.8%), stepfamilies (11.8%), or single-parent families (5.8%). Household income levels varied, but approximately one-third of families reported incomes below $30,000 (29.4%) and between $50,000 and $69,999 (29.4%). Half of the fathers (52.9%) and around 40% of the mothers (38.9%) had university degree. Most fathers were employed full time (77.7%), whereas half of the mothers identified as homemakers (50.0%). Finally, while the majority of parents were born in Canada (mothers 61.1%; fathers 44.4%), the remainder of the sample had immigrated from various countries in Africa (mothers 22.2%; fathers 27.8%), Europe (mothers 0%; fathers 11.1%), and Asia or the Middle East (mothers 11.1%; fathers 11.1%).

**Table 2:** Overview of family characteristics^a^.

Variables	Missing (%)	%		
Family situation	15.0			
Nuclear		70.6		
Separated or divorced		11.8		
Stepfamily		11.8		
Single		5.8		
Annual household income (CAN)	15.0			
$10,000–29,999		29.4		
$30,000–49,999		11.8		
$50,000–69,999		29.4		
$70,000–89,999		11.8		
$90,000–119,999		0		
$120,000+		17.6		
	Mothers		Fathers	
	Missing (%)	%	Missing (%)	%
Parents' level of education	10.0		15.0	
High school or lower		33.3		17.7
DCS/DVS^b^		27.8		29.4
University		38.9		52.9
Parents' employment status	9.0		9.0	
Full-time, salaried employee		33.3		77.7
Part-time, salaried employee		5.6		5.6
Homemaker		50.0		11.1
Student		11.1		0
Other (on leave, retired)		0		5.6
Parents' place of birth	9.0		9.0	
Canada		61.1		44.4
Central or South America		5.6		5.6
Africa		22.2		27.8
Asia and Middle East		11.1		11.1
Europe		0		11.1

a Participants provided information about their non-participating spouse.

b In Québec, a diploma of college studies (DCS) is a postsecondary degree in preparation for university-level education or a trade; adiploma of vocational studies (DVS) is a secondary degree in preparation for a specialized occupation.

At the beginning of the program, their child with DD was on average 4 years old (from 8 months to 6.5 years). Some children were awaiting a diagnosis (36.8%). Autism diagnoses were the most frequent (26.3%), followed by global developmental delay (10.5%), language delay (10.5%), and other diagnoses (e.g., chromosomal anomaly, genetic syndrome, cerebral palsy or other neurological disorder). On average, children had received this diagnosis 11 months (from 1 week to 1.5 year) prior to the start of E-PAtS. None of the families had received any intervention or support services prior to E-PAtS.

### 2.4 Measures: research team

#### 2.4.1 Program adaptation

##### 2.4.1.1 Adaptation log

An adaptation log was used to track the process, steps, and changes involved in translating and adapting the program to address Question 1. This process included the translation of the program materials into French and adaptations made to materials, content, inclusion or exclusion criteria, and delivery methods based on local needs and cultural factors in the first translation phase (i.e., before the delivering of the intervention in French) and for each cohort. The principal investigator (first author) and two research coordinators (second and third authors) maintained this log throughout the project.

#### 2.4.2 Feasibility and social validity evaluation: observation measures

##### 2.4.2.1 Attendance logbooks

A research assistant was tasked with observing each intervention group and using a logbook to capture the following aspects of intervention feasibility: absences, late arrivals, and the reasons provided for these. This information offered insights into attendance patterns, adherence to the program, and levers and barriers to consistent participation according to observers, facilitators, and participants.

##### 2.4.2.2 Observation logs

Observers noted parents' comments and reflections regarding the program to obtain insights on its social validity according to them and to the facilitators. Key events that influenced the course of the intervention according to participants and facilitators were also documented. After each session, the observer and facilitators engaged in debriefing to revise and complete the observation logs.

##### 2.4.2.3 Fidelity checklist

The E-PAtS manual includes a fidelity checklist designed to monitor the implementation of the program. This checklist outlines the themes included in each group session and is typically self-completed by facilitators. In the current study, observers used the checklist in real time to track the thoroughness of session content coverage (program curriculum: topics, activities, materials, and underlying theory) and co-delivery dynamics (i.e., which facilitator presented the material).

### 2.5 Measures: family carers

#### 2.5.1 Feasibility and social validity: semi-structured interviews

Participants' experience and perceptions were documented in three semi-structured interviews: before the program (pre-interview), immediately after the program (post-interviews 1), and three months later (post-interview 2). The topics covered in these interviews addressed several aspects of intervention feasibility and social validity.

##### 2.5.1.1 Pre-interview

The pre-interview guide was developed by the U.K. team as part of the E-PAtS curriculum process. It aims to prepare participants by providing clear information about the program. It also seeks to identify participants' specific needs and potential barriers to their participation (e.g., reading difficulties, cultural differences) to ensure that the program is aligned with their expectations. The present study specifically extracted participants' reasons for attending the program to analyze the alignment between program objectives and parents' needs.

##### 2.5.1.2 Post-interviews

The post-interview guides were co-developed and refined with input from an expert committee (external to the intervention) comprised of clinicians, researchers, and parents. This guide aims to collect information on the social validity of the program, specifically the adequacy of its goals for families' needs, the acceptability of its procedures, and its perceived effects on family members. These interviews also documented parents' perceptions on barriers to, and facilitators of, participation in the E-PAtS intervention.

#### 2.5.2 Social validity: standardized outcome measures

Questionnaires assessing the acceptability of procedures and perceived effects of the program on children and parents, two aspects of social validity (19), were selected based on the following criteria: 1) alignment with the desired outcomes outlined in the logic model for E-PAtS (20), 2) strong psychometric properties, 3) consistency with the original U.K. E-PAtS studies, 4) French-language validation with the target population. One questionnaire focused on perceived group cohesion, a central component of the E-PAtS approach. Three questionnaires were used to assess parents' psychological well-being, mental health, and sense of competence; one questionnaire was used to assess children's socioemotional and challenging behaviors.

##### 2.5.2.1 Group Cohesion scale

The revised Group Cohesion Scale [GCS-R; (28)] is an 8-item questionnaire (4-point Likert scale) designed to measure the perception of members within the same group on several aspects of group cohesion including: interaction and communication, member retention, decision-making, vulnerability between group members, and the alignment between group and individual goals.

##### 2.5.2.2 Warwick-Edinburgh mental well-being scale (WEMWBS)

This 14-item scale evaluates parents' overall well-being (a primary outcome in U.K. studies on E-PAtS) by measuring key elements of positive psychological functioning including affect, thoughts, optimism, and confidence. The presence or frequency of measured aspects is rated on a 5-point Likert scale (29).

##### 2.5.2.3 Hospital anxiety and depression scale (HADS)

This 14-item questionnaire assesses parents' symptoms of depression and anxiety through its two 7-item subscales. Symptom severity is rated on a 4-point Likert scale (30).

##### 2.5.2.4 Parenting Sense of Competence Scale (PSOC)

This 16-item questionnaire measures parents' self-perceived competence in their parenting role. It consists of two separate subscales that measure efficacy and satisfaction. Items are rated on a 6-point Likert scale (31).

##### 2.5.2.5 Developmental behavior checklist (DBC)

The 96-item questionnaire assesses social-emotional and behavioral problems in young children with DD. Items are rated on a 4-point Likert scale (32).

### 2.6 Procedure

#### 2.6.1 Training

All Quebec team members completed the five-day E-PAtS facilitator training delivered online by the lead developers of the program (NG and JB). Collaborators from the four clinical partner sites of the study joined as practitioner-facilitators and assisted in recruiting parent-facilitators. The initial group of facilitators consisted of three family carers, a social worker, a psychologist, an administrator (who was also a psychologist and researcher), along with three university researchers and a doctoral student. After conducting their first group session, three facilitators [one practitioner (psychologist) and two family carers] received additional training, also provided by NG and JB, to enable them to subsequently train other facilitators in Quebec. For this adaptation study, they trained five new facilitators (two family carers and three psychologists) to implement E-PAtS.

#### 2.6.2 Recruitment

The preliminary testing of E-PAtS took place between the fall of 2020 and the summer of 2022, a period spanning multiple waves of quarantine measures associated with Covid-19. The pandemic situation disrupted several services and created unanticipated challenges in accessing comprehensive family data. It was therefore not possible to determine the exact number of eligible families at the four participating centers. Consequently, a recruitment target of six cohorts of six families (36 families) was set. Families facing specific risk factors, such as sociodemographic or psychological risk indicators, were prioritized for inclusion in the study. Each partner center followed a predefined procedure for participant recruitment. The center first identified a practitioner to be paired with a parent to form the facilitator pair and established a schedule for delivery of the program. A clinical coordinator was responsible for screening the parent waitlist to identify those who met the inclusion criterion for the program, i.e., having a child aged under the age of 7 who was diagnosed with DD or awaiting diagnosis. This person contacted eligible parents to introduce E-PAtS and the research project. Parents who were interested and available to participate in the upcoming cohort as scheduled made an appointment with a research assistant to further discuss the project and sign the consent form. Of the 36 families targeted, 31 expressed interests and gave their consent to participate in both the program and the research project. Although both parents were invited to participate, as described in the Participants section, only one parent per family ultimately took part in the intervention.

##### 2.6.2.1 Data collection

The principal investigator and two research project coordinators recorded all meetings, decisions, and actions taken to document the translation and adaptation process. Prior to their participation in the 8-session program (T1), the practitioner-facilitator conducted individual pre-interviews with family carers as part of the typical E-PAtS process. These preliminary meetings were usually scheduled one to two weeks before the first group session. A research assistant also emailed parents the first set of parent and child questionnaires (outcome measures) to be completed before their first group session. Parents could request the assistance of the research assistant to complete these forms. As described in Table 1, an initial 30- to 45-min group meeting was added to the remote adaptation of the program to guide parents in using the online learning platform and videoconferencing software. The weekly intervention sessions were then delivered virtually by the co-facilitators. At the conclusion of the two months (8 sessions) of the program, a research assistant emailed parents the T2 questionnaires and scheduled their T2 post-interviews. Data collection for T2 was completed within two weeks following the last session. This process was repeated three months later (T3) with the same research assistant. Parents received a CAN$20 gift card upon completion of the questionnaires and interviews at each time point.

To enable the E-PAtS facilitator pairs to focus on program delivery rather than research procedures, research assistants were responsible for recording feasibility and social validity data (see Measures: Research Team) without intervening. These observers received preliminary training and were provided with examples of relevant information to record. Participants provided consent for the observer to take detailed notes throughout each session.

The post-program, semi-structured interviews with parents were conducted by the trained doctoral (Ph.D.) students in psychology. These interviewers had no rapport with the participants and had not yet interacted with them nor engaged with the program content itself. The interviews for the first cohort were conducted by the research coordinator (second author). Interviews for subsequent cohorts were carried out by other doctoral students who were not involved in the program's implementation. To mitigate the risk of bias, all interviewers followed the standardized interview guide and received extensive training as well as ongoing supervision by the research team (specifically, the first two authors).

### 2.7 Analyses

#### 2.7.1 Quantitative analysis

Descriptive statistics (frequencies, means, and standard deviations) were computed to summarize sociodemographic variables and trends in parent and child outcome measures. Because the goal of this step in the research process was to determine whether outcome variables fluctuated in the desired directions over the course of the intervention, these analyses are descriptive rather than inferential [i.e., they do not rely on statistically significant changes; see (33)].

#### 2.7.2 Qualitative analysis

Notes from pre-interviews were extracted and organized using spreadsheet software. These were categorized through a content analysis approach that focused on the following themes: 1) participant expectations for the program, 2) family experiences and services trajectories, 3) participant-perceived needs related to services.

Post-interviews were transcribed and imported into the MAXQDA software (34). Interview transcripts were analyzed qualitatively using the systematic content analysis method developed by L'Écuyer (35). This analysis employs both deductive and inductive approaches in two successive stages. In the first stage, content was categorized into 1) intervention feasibility aspects (barriers, facilitators) and 2) the three components of social validity (i.e., adequacy of the goals, acceptability of procedures, and perceived effects). In the second stage, the principal investigator and a research assistant conducted an initial reading of the transcripts to identify themes corresponding to these two topics. They each independently developed an initial coding grid consisting of themes and subthemes, which they subsequently combined and refined through discussion. They applied this revised grid to code units of meaning (UM) from two transcripts. UM are segments of responses expressing a full idea or specific action relevant to a theme. Next, two authors and four trained students used the grid on all transcripts. Their feedback led to additional adjustments to the themes and subthemes. All transcripts were then re-coded with the final grid. Results were reviewed until consensus was reached (48).

## 3 Results

This section outlines the steps, changes, and processes involved in the translation and adaptation of E-PAtS to the context of Quebec's public services and summarizes data on the intervention's feasibility and social validity based on feedback from the participating family carers.

### 3.1 Reactive program adaptations

The adaptation of E-PAtS for Quebec was iteratively refined across the six consecutive cohorts. These changes were informed by facilitators' experiences, observers' notes, and parents' recommendations shared in their post-interviews. The Covid-19 pandemic also prompted continual revisions to the program. At the close of each cohort's 8-week program, discussions among the CBPR team guided the development of the enhanced version.

#### 3.1.1 Content changes

After Cohort 1, videos from the original program were no longer included in sessions. These had not been translated and the live translation by program facilitators was not perceived as helpful by parents. The decision to remove these videos was prompted by a noticeable drop in parents' attention and interest when watching the clips. The videos were replaced by personal examples shared by the parent-facilitator. Across the first two cohorts, more than half of parents recommended adding more content on available services and on the pathway to accessing these services (e.g., the steps from diagnosis to specialized services, who to contact, transitions between services).

#### 3.1.2 Delivery changes

Parents in Cohort 1 expressed a desire for easier access to materials. As a result, the team discontinued its use of the online learning platform to share materials (Moodle). Subsequent cohorts received handouts and links to videoconferencing sessions via email. After Cohort 5, the decision was made to return to a format up to 12 families per group, as suggested in the original in-person E-PAtS intervention, for online delivery.

### 3.2 Intervention feasibility

#### 3.2.1 Retention rates and adherence to the program

All 31 parents who participated in the program and research project completed the pre-interview with a facilitator. Six parents withdrew before the first group session, one after the second session, two after the third session, and one after the fourth. The two main reasons cited for withdrawal were a lack of time and the delay (often several weeks) between their recruitment and the start of the E-PAtS groups, during which interval some participants' schedule and availability had changed. Of the remaining 21 participants, 17 attended all sessions, two missed only one session, and two missed two. Parents reported being unable to attend sessions due to medical appointments (*n* = 3), technical issues (*n* = 1), childcare issues (*n* = 1), illness (*n* = 1), or work commitments (*n* = 1). Table 3 lists attendance details for each cohort.

**Table 3:** Session attendance by each cohort.

Session	Cohort 1 (*n* = 4)	Cohort 2 (*n* = 5)	Cohort 3 (*n* = 5)	Cohort 4 (*n* = 5)	Cohort 5 (*n* = 2)	Cohort 6 (*n* = 4)	Total (*n* = 25)
1	4	4	4	5	2	4	23
2	4	4	5	5	2	4	24
3	4	4	5	5	2	4	24
4	4	4	4	3	2	4	21
5	4	4	4	3	2	4	21
6	4	4	3	3	2	4	20
7	4	4	3	3	2	4	20
8	4	4	2	3	2	4	19

The *n* for each cohort includes participants who withdrew during the program but excludes those who dropped out before Session 1. Cohort 1 took place at a community-based evaluation clinic. Cohorts 2, 5 and 6 were organized by public health and social services agencies. Cohorts 3 and 4 took place at a specialized pediatric hospital.

Although both parents (or other family carers) in each family were invited, due to availability and scheduling challenges, only one parent (most often, the child's mother) from each family was able to enroll in the program. However, 91% of parents said that they shared content and information learned during the sessions with their partner. Four participants also mentioned sharing knowledge with extended family members or other significant individuals in the child's life.

#### 3.2.2 Perceived facilitators of E-PAtS participation

In response to the post-interview questions on facilitators to participation, respondents identified four elements that supported their involvement in the program: 1) online session delivery (e.g., not needing to travel; 9 parents), 2) having someone to look after the children at home [4 parents, “he looked after the kids while I did it so it was kind of my thing, you know (mother 3 in cohort 1); 3] a bond of trust between parents and facilitators [2 parents, “I think everyone in the group had that, they were very comfortable with everything (mother 3 in cohort 1″); and 4] small group size [1 parent, “And I think it helped that […] there were only four parents (mother 3 in cohort 1″)”].

Three parents spontaneously mentioned that in-person delivery would have facilitated sharing personal experiences and fostered engagement in the program. As one parent noted, “I think we could do more activities; we could share more […] even bring […] our children and do exercises with them (mother 2 in cohort 6).”

#### 3.2.3 Perceived barriers to E-PAtS participation

In the post-interviews, participants were asked about barriers to their participation in the program. They identified six major obstacles: 1) parents' mental or general health difficulties [6 parents, “[…] with three little ones, you know, often in the evening I'burned out (father 2 in cohort 3)”]; 2) scheduling conflicts [4 parents, “For a parent who doesn't have this flexibility, who doesn't have an office job, that would be impossible (father 2 in cohort 4)”]; 3) children's health issues [4 parents, “There were two sessions that I didn't attend […] when I went to the hospital with my daughter (mother 5 in cohort 5)”]; 4) having to look after their children at home while attending the sessions [3 parents; “you know, in the evening, that's the time I now devote to the children (mother 2 in cohort 1)”]; 5) the timing of sessions (1 parent); and 6) lack of technical proficiency with the videoconferencing software (1 parent).

#### 3.2.4 Recommendations for future delivery

Participants were asked about, and also spontaneously shared, their recommendations for future E-PAtS groups. These suggestions are listed in Table 4.

**Table 4:** Participants' recommended changes for future delivery (*N* = 14).

Type of changes	*n*	UM	Sample quote
Content			
Increase coverage of services available to parents	7	16	“Maybe we need to talk about it, and this is just my opinion: instead of doing one session, do two sessions so that people understand their rights and their needs”
Spend more time on some topics	6	8	“I don't know if there's anything more to say than what you shared, but sleeping, sleeping can be such a huge problem for the kids, I think my kids specifically, so…”
Add a final post-program meeting	6	9	“That we can see these same people again, you know, maybe three months later, then six months later, you know, to see how things are progressing.”
Further explain the trajectory of services	3	5	“It would be good if there was information about when they are going to school and the options and also, like, some clear information about […] cause I only heard this stuff through friends”
Delve deeper into intervention strategies for behaviors that challenge	2	3	“Maybe bring some, some ideas like that to the level of tantrums. Like, broaden it more. Maybe more small interventions that come from real experience there, too.”
Develop new content (siblings)	1	1	“The impact on siblings, you know how we didn't talk about it, because we brought it up but it wasn't in the basic content.”
Delivery			
Ensuring group families have children with similar profiles	8	22	“It would be easier if families were grouped according to the particularities of their children and also by age.”
Improving access to material	5	12	“I think that for the handout, next time I think it would be good if you have the possibility to send it to participants before the start [of the program].”
Providing more sessions, with the same duration	3	4	“We would have liked the program to continue longer!”
Providing more sessions, with a shorter duration	1	4	“Honestly, I think the sessions could have been shorter. But over a longer period…”
Keeping a small number of participants per group	1	1	“And if there [were] more [parents], definitely we wouldn't all get to speak […] or say much, I think”
Finding strategies to facilitate participation of both family carers	1	1	“But if [there] were more fathers, it would be more easier for me [to share].”
Being offered the choice of online or in-person delivery	1	1	“It would be to offer both. Like offering a, like a zoom opportunity and an in-person opportunity.”

The *n* represents the number of parents who discussed this theme in either of the two post-interviews. UM, Units of meaning.

##### 3.2.4.1 Content

Seven parents suggested that more time should be spent on the services to which families are entitled, e.g., local resources or financial support. Three parents mentioned that it could be useful to include more information on the trajectory of services for families of children with DD within public health and social services. Several parents suggested planning for a post-program meeting for participants (*n* = 6). Six parents expressed a desire for more time to discuss certain topics (e.g., sleep). Two parents mentioned wanting to delve deeper into interventions strategies for behaviors that challenge and have more specific examples based on real-life situations. One parent proposed adding content focused on siblings.

##### 3.2.4.2 Delivery

More than half of the participants who completed the program (*n* = 13) made recommendations about the delivery of E-PAtS. Eight parents recommended to group together families whose children have similar needs or profiles. Five parents proposed improving access to materials (the material was provided through the Moodle platform, which was not adequate according to parents). Some parents requested more sessions of either the same duration (*n* = 3) or shorter durations (*n* = 1). One parent mentioned that it would be desirable to offer a choice of online or in-person delivery. One parent suggested to keep the number of parents in the group low because more attendees could have decreased the willingness to participate. A parent suggested to find strategies to facilitate attendance by both family carers.

#### 3.2.5 Intervention fidelity

##### 3.2.5.1 E-PAtS Curriculum

Data from the fidelity checklist are displayed in Tables 5, 6. On average, 89.6% of the program content was covered for five of the cohorts (the fidelity checklist was not available for Cohort 1). Globally, the first session, *Working Together,* had the lowest fidelity scores (73%), while Session 6, *Responding to challenges—Part 1,* had the highest (95%). Group 2 had the lowest fidelity score and Group 3, the highest.

**Table 5:** Intervention fidelity: curriculum content coverage With each cohort.

Cohort	Content covered (%)								
	Session 1	Session 2	Session 3	Session 4	Session 5	Session 6	Session 7	Session 8	Total
2	51.35	88.00	86.11	76.67	76.00	90.00	88.89	100.00	82,13
3	83.78	100.00	94.44	100.00	96.00	100.00	96.30	100.00	96,32
4	72.97	88.00	91.67	NA	100.00	93.33	81.48	83.33	87,26
5	78.38	96.00	88.89	100.00	100.00	96.67	100.00	NA	94,28
6	78.38	96.00	88.89	100.00	100.00	96.67	100.00	50.00	88,74

The fidelity checklist was missing for the first group; NA, Not available.

**Table 6:** Intervention fidelity: session Co-facilitation.

E-PAtS sessions	Content covered by facilitators (%)			
	Practitioner	Parent	Both	Total
Session 1—Working together	28.6	19.5	24.9	73.0
Session 2—Looking after you and your family	30.4	34.4	28.8	93.6
Session 3—Sleep	42.2	32.2	15.6	90.0
Session 4—Interaction and communication	41.7	30.0	22.5	94.2
Session 5—Fostering life skills through active development	40.0	44.8	9.6	94.4
Session 6—Responding to challenges—Part 1	49.3	30.7	15.3	95.3
Session 7—Responding to challenges—Part 2	40.7	29.6	23.0	93.3
Session 8—Bringing it all together	29.2	29.2	25.0	83.3
Total	37.8	31.3	20.6	89.6

The fidelity checklist was missing for the first group.

##### 3.2.5.2 Co-facilitation

As displayed in Table 6, there was good balance between the two co-facilitators' engagement in content coverage across sessions. Practitioners were more involved (10%–20% more engagement) in five of the sessions, whereas parents took a more active role in Session 2 (*Looking After You and Your Family*) and Session 5 (*Fostering Life Skills Through Active Development*).

### 3.3 Social validity

The observers' notes taken during sessions and data from the two post-interviews captured parents' insights regarding the three components of social validity as detailed below. In addition to the post-interviews, the outcome questionnaires that parents completed yielded social validity data regarding perceived intervention effects.

#### 3.3.1 Adequacy of goals

All participants reported that the program was consistent with how E-PAtS had been described to them and aligned with why they chose to participate. Ten parents said they enrolled in the program to receive formal support and learn intervention strategies. Eight participants indicated that they sought opportunities to discuss and share with, and learn from, other parents in similar situations. In this regard, a parent said: “What I find most important is allowing everyone to share how they experience their challenges with their children (father 3 in cohort 3).” Five parents were motivated to participate because a practitioner they trusted had recommended it to them. Finally, one parent also wanted to validate their parental practices.

Parents also shared ways in which the goals of the program met their needs. Eight stated that the intervention focused on enhancing parental well-being: “the emphasis was on the fact that it was a group that was going to be more for the well-being of parents (mother 2 in cohort 1).” Five parents also thought the program aimed to address how to manage the child's behavior. One parent mentioned E-PAtS' goal of providing information about existing services.

#### 3.3.2 Procedure acceptability

Table 7 presents the results of the qualitative analysis of parents' comments on the acceptability of E-PAtS components. Twelve subthemes related to aspects that parents valued the most; seven discussed what they liked least. Because these themes and subthemes were spontaneously generated by parents, a lower percentage does not imply that other parents did not also appreciate a given aspect, only that it was not spontaneously mentioned during their interviews.

**Table 7:** Social validity: acceptability of procedures.

Themes	Subthemes	T2 post-interview		T3 post-interview	
		*n* (%)	UM (%)	*n* (%)	UM (%)
Strenghts		14/14 (100)	346/450 (76.9)	10/10 (100)	45/74 (60.8)
	Session content and themes	14/14 (100)	49/346 (14.2)	1/10 (10.0)	1/45 (2.2)
	Co-facilitation by parent and professional	14/14 (100)	57/346 (16.5)	7/10 (70.0)	9/45 (20.0)
	Material	14/14 (100)	40/346 (11.6)	1/10 (10.0)	2/45 (4.4)
	Online delivery format	12/14 (85.7)	27/346 (7.8)	3/10 (30.0)	3/45 (6.7)
	Overall assessment of the program	12/14 (85.7)	27/323 (8.4)	7/10 (70.0)	8/45 (17.8)
	Parent support group, safe space for sharing	12/14 (85.7)	72/346 (20.8)	8/10 (80.0)	15/45 (33.3)
	Session duration	11/14 (78.6)	14/346 (4.1)	0	0
	Activities and exercises	11/14 (78.6)	25/346 (7.2)	2/10 (20.0)	4/45 (8.9)
	Delivery schedule	8/14 (57.1)	9/346 (2.6)	0	0
	Program content alignment with family needs	6/14 (42.9)	9/346 (2.6)	0	0
	Program duration	5/14 (35.7)	5/346 (1.5)	1/10 (10.0)	0
	Program flexibility	3/14 (21.4)	4/346 (1.2)	0	0
Areas of improvements		12/14 (85.7)	27/450 (6.0)	2/10 (20.0)	2/74 ()
	Access to materials (online)	5/14 (35.7)	8/27 (29.6)	1/10 (10.0)	1/2 (50.0)
	Online delivery format	5/14 (35.7)	8/27 (29.6)	1/10 (10.0)	1/2 (50.0)
	Program duration	5/14 (35.7)	7/27 (25.9)	0	0
	Delivery schedule	1/14 (7.1)	1/27 (3.7)	0	0
	Diversity of family needs	1/14 (7.1)	1/27 (3.7)	0	0

Proportions refer to the number of parents who spontaneously mentioned a given theme.

##### 3.3.2.1 Strengths

All parents mentioned that they appreciated the content and themes of the sessions as well as the fact that E-PAtS was delivered by co-facilitator dyads consisting of a parent and a practitioner. For instance, a parent said: “I found that they [the two facilitators] were respectful, knew what they were doing, and worked well together. There was strong collaboration between them. (father 3 in cohort 3)” A large number of parents appreciated the online and support group format of the program. Other positively regarded elements included the duration and scheduling of sessions, program materials, and activities.

##### 3.3.2.2 Areas for Improvement

The elements that parents found that needed improvement comprised three subthemes: the accessibility of materials (especially handouts) on the online learning platform (Moodle), the online format (i.e., these parents would have preferred in-person sessions), and the program duration (i.e., more sessions were needed). Thus, these elements related primarily to technical aspects of intervention delivery rather than the general approach and content of the program.

#### 3.3.3 Perceived effects

Table 8 summarizes the major themes identified in parents' discourse on the perceived impact of E-PAtS: 1) parental knowledge and skills, 2) parental well-being, and 3) community and material resources.

**Table 8:** Social validity: perceived effects of the E-PAtS program discussed by parents.

Themes	Subthemes	T2		T3	
		*n* (%)	UM (%)	*n* (%)	UM (%)
Parental knowledge and skills		10/11 (90.9)	39/66 (59.1)	9/10 (90.0)	27/63 (42.9)
	Parental practices, self-confidence and sense of competence	9/10 (90.0)	24/39 (61.5)	8/10 (80.0)	13/27 (48.2)
	Related to child's needs and development	7/10 (70.0)	15/39 (38.5)	7/10 (70.0)	14/27 (51.9)
Parental well-being		7/11 (63.6)	19/66 (28.8)	4/10 (40.0)	10/63 (15.9)
	Self-care and mental health	5/7 (71.4)	13/19 (68.4)	4/4 (100.0)	9/10 (90.0)
	Social validation, normalization, and reduced feelings of guilt	4/7 (57.1)	6/19 (31.6)	1/4 (25.0)	1/10 (10.0)
Community and material resources		5/11 (45.5)	5/66 (7.6)	7/10 (70.0)	19/63 (30,2)
	Development of a mutual aid system	3/5 (60.0)	3/5 (60.0)	5/7 (71.4)	15/19 (78.9)
	Preparation to seek and use other services	2/5 (40.0)	2/5 (40.0)	2/7 (28.6)	2/19 (10.5)
	Development of empathy for other parents and families	0	0	2/7 (28.6)	2/19 (10.5)

*Note.* The *n* represents the number of parents who mentioned a given theme or subtheme. UM, unit of meaning.

Ten parents shared that E-PAtS helped enhance their abilities in, and perceptions of, their roles as parent or their knowledge of their child's needs and development. For instance, one parent said: “[I learned that] he's trying to tell me that he wants to do this and that, but he doesn't know how, he's not just complaining about it. (mother 3 in cohort 1)”. Seven parents discussed effects on their well-being such as an improvement in their self-care abilities and the recognition of the importance of their mental health. A mother said: “[it] was really good, in E-PAtS, to discover how to take care of yourself, how to take the time, even if it's just for a coffee, […], to look out the window or do activities. I put myself first now. (father 4 cohort 5)”. Relatedly, parents shared how E-PAtS showed them that their experiences were valid and normal, which helped to alleviate feelings of guilt. Finally, E-PAtS helped parents find informal and formal support resources. For instance, for the informal support, they appreciated the mutual aid system, being part of a group and agreeing to help each other in the future. On the formal support system, a parent shared: “[the idea is] to insist, not just make a request and then not follow up, to insist on the subject. I did obtain a psychoeducator who takes care of my daughter. (father 3 in cohort 3)”.

Table 9 displays scores on parent and child outcome measures collected before the program (T1), at the end of the program (T2), and three months later. With one exception (anxiety), these measures presented trends over time that were consistent with program goals and parents' testimonials, namely increases in parents' well-being and sense of competence and a decrease in parents' depression and children's behavioral and emotional challenges. However, anxiety, which exceeded the clinical threshold in 65% of respondents at T1, remained fairly stable. Finally, parents reported a high perception of the group cohesion (between 3.8 and 4; *M* = 3.9, SD = 0.13).

**Table 9:** Social validity: perceived effects documented through parent and child outcome measures.

Measures	T1		T2		T3	
	M	SD	M	SD	M	*SD*
Parent outcomes						
Well-being (WEMWBS)	49.5	11.5	52.0	8.7	52.0	10.8
Anxiety (HADS)	11.8	3.3	11.8	2.7	12.0	2.9
Depression (HADS)	9.2	2.2	8.1	1.8	7.7	2.5
Sense of competence (PSOC)	78.3	10.8	83.0	13.0	83.1	11.6
Child outcomes						
Challenging behavior (DBC)	75.2	32.6	69.3	38.9	68.8	48.7

T1 = immediately before the program, T2 = immediately after the program, T3 = three months after the program. WEMWBS, Warwick-Edinburgh Mental Well-Being Scale; HADS, Hospital Anxiety and Depression Scale, PSOC, Parental Sense of Competence, DBC, Developmental Behavior Checklist.

## 4 Discussion

In response to a clinical context marked by excessive waiting times, inequities in access to early intervention, and a lack of psychological support for family carers within Quebec's public health and social services system, the E-PAtS program, originally developed in English and tested in the United Kingdom (20), was selected and adapted for French-speaking families of children aged 0–6 years 11 months years who are suspected of, or diagnosed with, a DD. Implementing an intervention in a new context, especially where linguistic and services system differences exist, requires its tailoring to integrate local specifics and carefully testing its feasibility and acceptability within this new setting (23). Adaptation studies are therefore important steps toward larger evaluations of an intervention's efficacy in a new context. This article describes the steps and processes used to adapt E-PAtS for implementation within Quebec's public health and social services. The resulting Canadian French program is the product of a collaboration between the team who originally developed the intervention in the U.K. and a Canadian research team. Representatives from all stakeholders' groups (i.e., parents, practitioners, administrators, and researchers) participated in the iterative testing and continuous refinement of the program over six successive cohorts. This process was dynamic rather than linear, as lessons learned were continuously integrated as the project progressed.

### 4.1 Co-translation of E-PAtS to french and its co-adaptation for Quebec services

The first step in making the E-PAtS intervention feasible within Quebec's social and health public services was to build a CBPR team to modify the program for this new linguistic, cultural, and clinical context. This entailed the adaptation and translation of training and intervention materials to Canadian French in preparation for future trial studies and clinical implementations. These changes were made collaboratively by family carers, clinicians, administrators, and researchers to ensure the implementation of a realistic and socially valid program that could be incorporated quickly and effectively into real-life practice settings. This stage was critical not only for the practical feasibility of the intervention but for the actualization of key CPBR principles [see (19)]. Specifically, it facilitated a co-learning process and established the foundations of a lasting partnership among stakeholders, empowered family carers and clinical teams, and set the stage for the research design to be used in future evaluation and deployment stages. This stage allowed us to reflect as a team on how to involve everyone in the research process and ensure that this and future planned studies aligned with families' and service providers' values and needs.

The support of the E-PAtS program developers was a major lever in the translation and adaptation phase. Rather than focusing on strict adoption, the program proactively anticipated the need for adaptations. Local adaptations had already been tested across different regions of the U.K. (e.g., Northern Ireland, Scotland, England). This study is the first adaptation outside of the U.K. and in another language than English. The U.K. team offered very important guidance on making changes to the program or its manual while also focusing on the core underlying theory of the program and the mechanisms of change that should remain consistent.

### 4.2 Tailoring and preliminary testing of the intervention in real-life settings

The four preliminary testing sites for the adapted E-PAtS program were selected to represent the diversity of Quebec's administrative regions (i.e., urban and rural) and of establishments that serve families (i.e., from regional public agencies to specialized pediatric hospitals). This deliberate selection of participating centers enabled us to observe a diverse range of implementation conditions and assess how the program could be applied across different geographical areas and population characteristics. This was also a test of the program's adaptability and effectiveness in meeting the needs of diverse communities and settings. With the aim of building capacity and ensuring intervention sustainability in the partner agencies, we planned the training and supervision system for E-PAtS facilitators directly within these establishments. We sought to ensure the long-term viability and success of the program, independently of future research initiatives, by focusing on strengthening the skills and knowledge of facilitators within their clinical environments. These decisions reflected our team's core values and ensured that the program remained relevant, acceptable, and impactful for a representative cross-section of the targeted population and that it was suited to the realities and resources of the public system.

### 4.3 The COVID-19 pandemic

This project was initiated before, but launched during, the Covid-19 pandemic. The Quebec team was trained for E-PAtS in February 2020, just before the March 2020 lockdown measures were implemented in the province. This prompted a brief interruption of the project in order to adapt the intervention delivery format and several aspects of the research design accordingly. These changes impacted our deployment and evaluation plan as well as the results in several ways.

First, delivery of E-PAtS was postponed due to a wide-scale lockdown and the interruption of all governmental services in Quebec, including those provided by the partner sites. Services were restored slowly in September 2020, but the personnel members who had been deployed in pandemic response units only returned gradually to their normal roles. This prolonged disruption in normal staffing slowed the progress of E-PAtS implementation and required some changes to the number of facilitators trained in the clinical settings and the timing of their involvement in the project. Once the partner sites had resumed their normal operations, the project timeline experienced further disruptions stemming from employees' and families' absence due to illness. Rather than pause the project until implementation conditions improved, we continuously adapted the intervention and searched for better ways to meet the changing needs of our partners and families.

Second, as illustrated in several studies, the pandemic context generally exacerbated risk factors for parents' mental health difficulties, which were already strained by the manifestations of their child's diagnosis and issues related to access to services (36, 37). Families, cut off from their formal and informal supports, experienced higher levels of distress (38). The high scores on measures of anxiety and depression symptoms in the present study suggest that many parents may have been experiencing such difficult situations. Although parents were unanimous in their appraisal of the positive effects of the program on their own adjustment, it is likely that many would have benefited from intensive and individualized support, as well respite care and material assistance, etc. These difficulties may have influenced the perceived effects of the program on mental health outcomes.

### 4.4 Recruitment and retention

Out of the 36 families invited to participate in the program, 31 families consented to take part in the intervention, and 21 completed at least five out of the eight sessions, which is the adherence criterion for E-PAtS. Even under challenging conditions, more than half of the families (17 family carers) attended all 8 sessions. Despite six parents withdrawing before the first session and especially given the pandemic context and well-documented dropout rates in group programs for parents [see (39)], this level of participation was deemed good. This underscores the program's significance, value, and relevance in families' service trajectories.

In this preliminary testing phase, only one carer per family (mostly the mother, 86%) was involved. The participation of a second family member is explicitly targeted in the program and expected to have an impact on family dynamics (20). Parents said they chose to prioritize the participation of one parent so the other parent could take care of the children, work, or attend medical appointments, all responsibilities that were made more difficult by the pandemic situation. However, many participating parents reported that they shared the program content with the second carer (typically, the father) or other family members, which suggests that E-PAtS could have an impact on the family even when only one family carer participates in sessions.

Participants mentioned several elements that facilitated their participation in the program, such as the online delivery format and their relationship with the facilitators and other parents. The partner sites' administrators and practitioners' commitment to supporting the E-PAtS program in spite of organizational challenges (i.e., disruptions to services, turnover, system-wide changes) and their dedication to finding solutions were also major facilitators for participant recruitment and retention.

### 4.5 Target population

The present project sought to identify mechanisms to reach families with diverse backgrounds and address social, economic, and diagnosis-related inequities in service accessibility and quality [e.g., (12–15)]. Participants varied in their sociodemographic characteristics. Notably, 40% of participants were from an immigrant background and 40% had a family income below the poverty threshold for the province. The sample included families of children with a range of DD diagnoses and, importantly, families whose child had yet to be evaluated. Thus, the E-PAtS program is poised to address diagnostic inequities by providing timely support to families based on their service needs and to mitigate inequities related to long waiting lists and delays in receiving a diagnosis and intervention. To better align with the organization of services within the Quebec public health system and to reach families before the critical transition to school (when specialized services for DD are typically discontinued), the program's target age range was expanded from 0 to 5 years to include children under age 7. This change was successfully implemented and deemed valid by the service providers (practitioners and administrators) and service users (parents).

### 4.6 Intervention fidelity

Intervention fidelity entails that the intervention is carried out as intended, including fidelity to the intervention materials (what was done), the quality of delivery (how it was done), and the amount or dose of the intervention received. According to data collected with the E-PAtS fidelity checklist, the program was implemented with high fidelity and high dosage. Session 1 (*Working together*) was the most difficult to cover with fidelity (73%). This session covers less content related to intervention strategies content but includes time for parents and facilitators to get acquainted and for parents to share their experiences and needs. The latter is an important goal of E-PAtS. Parents suggested that this session could be lengthened or converted in two sessions to allow for more time to share and for the facilitators to present information on available services. Session 6 (*Responding to challenges—Part 1*) had the highest fidelity score. Although two entire sessions (Sessions 6 and 7) are dedicated to the topic of behaviors that challenge, parents suggested that future implementations include more in-depth coverage of this subject. Behaviors that challenge directly impact family functioning, the psychological health of family members, and children's development (40) and are a high priority for intervention according to families (41).

With respect to the quality of delivery, engagement was well balanced between the two co-facilitators. Some sessions appeared to naturally elicit more involvement from professional facilitators (e.g., on behaviors that challenge) or from parent facilitators (e.g., parental well-being, and adaptive behavior). This co-delivery of the program by the facilitator dyad was an element that participants particularly appreciated and found impactful.

### 4.7 Adequacy and acceptability

Quebec presently lacks public programs specifically designed to support family adjustment and the psychological well-being of parents raising children with DD. Many parents have communicated that existing services fall short in addressing these critical needs (9, 42, 43). The participants of this study confirmed that the goals of the E-PAtS program of providing emotional support and practical solutions directly fill this gap. The content and strategies included in program address common challenges faced by families, such as behavior management and communication difficulties, without being restricted to specific diagnoses. The program incorporates strategies designed to motivate, engage, and empower parents. Families valued the materials, activities, and the structured format that provides space for sharing and discussions.

The co-delivery format of the program, which combines the professional expertise of clinical practitioners and the lived experiences of parents, was particularly appreciated by participants. This approach also sparked a change in practices in partner organizations, specifically the creation of an official parent facilitator role within the services. At present, the hiring of parents is not a common practice within network of public services in Quebec. Efforts are presently underway to align administrative processes with this innovative approach. This partnership models a respectful, enriching, and supportive approach to caregiving.

### 4.8 Perceived impacts

Parents unanimously said that the program had a positive influence on their life. They most often mentioned its impact on their parenting skills and knowledge, particularly with respect to intervention strategies in critical areas (e.g., behaviors that challenge, sleep, communication). They reported that the program helped them to provide more informed and effective support for their children's needs. This, in turn, increased their self-confidence and parental sense of competence. This latter improvement was observable in qualitative and quantitative data. These effects and the implementation of their new skills may have contributed to better outcomes for their children (as evidenced by behaviors that challenge ratings) and ultimately reduced stress and improved interactions in their family.

The majority of parents spontaneously reported an improvement in their well-being. Session 2 of the program (*Looking after you and your family*) equips family carers with practical tools and strategies to support their mental and emotional health, which is essential for sustainable caregiving. Parents shared that their self-care skills had been strengthened, and they gained a deeper understanding of the importance of their own mental health. These data demonstrate that this key objective of the program was successfully achieved. The quantitative mental health indicators presented mixed trends (i.e., increased well-being, decreased depression, but stable anxiety scores), which may be in part attributable to the context of the Covid-19 pandemic. It should also be noted that the questionnaire used in this study measured general anxiety rather than specific manifestations of stress and distress related to parenting practices in the context of DD. Future stages of program evaluation will include a more focused parenting stress indicator to better capture this aspect of caregiver well-being. In-depth measures of parents' emotional experiences throughout the program should also be considered [see, e.g., the addition of emotional journaling; (24)].

Parents noted that the group intervention format promoted the formation of supportive peer networks where family carers could exchange experiences and learn from each other. This emotional support helped to alleviate feelings of isolation and build a nurturing environment. Besides gaining knowledge about their child's development and intervention strategies, many parents said that one of the most valuable aspects of E-PAtS was its supportive and safe environment where they found a sense of belonging and mutual understanding. These findings align with the scientific literature that underscores the importance of informal peer support in family adjustment (44).

E-PAtS aims to build family and material resources across various dimensions through multiple processes. These processes were largely represented in the effects of the program as perceived by the parents. For instance, many parents reported improvements in their understanding of available services and that they were more inclined to ask for help and better prepared to search for and use other services. This empowerment helped them advocate for their children, communicate effectively with professionals, and access necessary resources efficiently.

### 4.9 Next steps in implementing E-PAtS within public services in Quebec

Beyond the present evaluation of E-PAtS' adaptation to a new cultural context, as previously described, the current study is a part of a larger CBPR initiative that aimed to co-evaluate and gradually implement the intervention within Quebec's health and social services system. As part of the planned sequence outlined in Figure 1, the next phase of this research will specifically evaluate the implementation of the adapted E-PAtS program. This evaluation will go beyond the adaptation of the model itself to systematically examine key implementation challenges, including issues of sustainability, feasibility, and acceptability by local service providers and decision-making bodies. Attention will also be given to the financial and organizational aspects of program delivery, which are essential for ensuring its long-term viability within the provincial system. The formal effects on parent, child, and family outcomes, its impact on program facilitators will be evaluated. The feasibility of conducting a future randomized controlled trial in such a setting will also be addressed. By documenting these dimensions, the planned study aims to comprehensively assess how the program can be successfully embedded into existing structures. Its results will inform strategies for sustainable dissemination and maximizing long-term impacts on families.

### 4.10 Limitations

The context of the Covid-19 pandemic prompted the adaptation of the program for online delivery. This is a significant positive aspect of this research that will enable the program to be offered even in remote areas. However, this change also means that the program could not be evaluated for in-person implementation as originally planned and that all research measures were administered online. In-person visits and interviews with parents may provide additional opportunities to assist them in completing questionnaires and sharing their rich and unique personal experiences. Face-to-face interactions may be instrumental in forming a close connection with parents, which is believed to have positive impacts on program participation and retention. Additionally, the program is open to two family carers (both the child's parents or a second adult carer). Adaptations to the program schedule or delivery may be needed to facilitate the participation of both carers despite the multiplicity of roles and responsibilities they share.

## 5 Conclusion

E-PAtS offers an innovative intervention that can reduce inequities caused by prolonged wait times for services and the social exclusion experienced by children with DD and their parents in the Quebec context. It addresses a lack of programs to support the psychological well-being of parents and fosters their empowerment, resilience, and ability to fulfill roles and responsibilities from the start of their journey. This, in turn, promotes the full development and social participation of their child. E-PAtS is designed to be low-cost and feasible for service providers and is applicable to various service contexts. The program also offers flexible delivery modalities (in-person as developed in the U.K., and online as implemented in this study). E-PAtS has the potential to ease families' experience throughout the remainder of their service trajectory. Partnering with parents, clinicians, administrators, and researchers is a core principle in the research process. The acknowledgment of parental expertise and strengths is one of the premises of this intervention. These values make the project more than research: it is a social initiative that recognizes that parents can participate in the development of interventions dedicated to them and their children in order to promote their quality.

## Data Availability

The raw data supporting the conclusions of this article will be made available by the authors, without undue reservation, through a request to the corresponding author.

## Footnotes

1 The term DD includes diagnoses, disorders, or conditions identified in early childhood that involve significant delays in key developmental domains, namely cognitive development, adaptive behaviors, motor skills, language, and social-emotional development. Examples of DD include diagnoses of autism, intellectual disability, and global developmental delay. However, some diagnoses, such as intellectual disability, are rarely given before school age. Various terms are used in the literature to define groups of delays, neurodevelopmental conditions, or atypical development in early childhood. In this article, DD includes children who have received an official diagnosis, those awaiting a diagnosis, and those with significant developmental delays or atypicalities that involve developmental support needs [for a discussion on terminology, see (3)].
